# Cannabinoid Receptor Type 2: A Possible Target in SARS-CoV-2 (CoV-19) Infection?

**DOI:** 10.3390/ijms21113809

**Published:** 2020-05-27

**Authors:** Francesca Rossi, Chiara Tortora, Maura Argenziano, Alessandra Di Paola, Francesca Punzo

**Affiliations:** 1 Department of Woman, Child and General and Specialist Surgery, University of Campania “Luigi Vanvitelli”, L. De Crecchio 4, 80138 Naples, Italy; chiara.tortora@unicampania.it (C.T.); punzofrancesca.phd@gmail.com (F.P.); 2 Department of Experimental Medicine, University of Campania “Luigi Vanvitelli”, S. Maria di Costantinopoli 16, 80138 Naples, Italy; maurargenziano@gmail.com (M.A.); alessandra.dipaola92@gmail.com (A.D.P.)

**Keywords:** SARS-CoV-2, cannabinoid receptor type 2, therapeutic strategies, molecular target, immune response, inflammation, endocannabinoids system, COVID-19

## Abstract

In late December 2019, a novel coronavirus (SARS-CoV-2 or CoV-19) appeared in Wuhan, China, causing a global pandemic. SARS-CoV-2 causes mild to severe respiratory tract inflammation, often developing into lung fibrosis with thrombosis in pulmonary small vessels and causing even death. COronaVIrus Disease (COVID-19) patients manifest exacerbated inflammatory and immune responses, cytokine storm, prevalence of pro-inflammatory M1 macrophages and increased levels of resident and circulating immune cells. Men show higher susceptibility to SARS-CoV-2 infection than women, likely due to estrogens production. The protective role of estrogens, as well as an immune-suppressive activity that limits the excessive inflammation, can be mediated by cannabinoid receptor type 2 (CB2). The role of this receptor in modulating inflammation and immune response is well documented in fact in several settings. The stimulation of CB2 receptors is known to limit the release of pro-inflammatory cytokines, shift the macrophage phenotype towards the anti-inflammatory M2 type and enhance the immune-modulating properties of mesenchymal stromal cells. For these reasons, we hypothesize that CB2 receptor can be a therapeutic target in COVID-19 pandemic emergency.

## 1. Introduction

SARS-CoV-2 (CoV-19) is a sense RNA virus with envelope- and spike-like projections on its surface [1]. It belongs to Coronavirinae family, whose genomes consist of about 30 kilobases, the largest genomes known among RNA viruses. Two-thirds of their genome encodes viral replicase/transcriptase functions that are involved in virus replication, while one-third encodes viral structural proteins and accessory proteins. Coronaviruses can infect a wide range of vertebrates including humans [2].

Prior to the outbreak of severe acute respiratory syndrome (SARS) in 2003, only two coronaviruses (hCoV-229E and hCoV-OC43) were known to infect humans. Following 2003, additional coronaviruses have been discovered in humans: SARS-CoV, hCoV-NL63, hCoV-HKU1, Middle East respiratory syndrome coronavirus (MERS-CoV), and the new SARS-CoV-2. SARS-CoV, MERS-CoV and SARS-CoV-2 are highly pathogenic in humans and cause severe acute respiratory distress with a high rate of mortality. Remarkably, all three viruses are believed to have originated from bats [3]. The latter, SARS-CoV-2, emerged in late December 2019 as responsible for a severe acute respiratory syndrome named COronaVIrus Disease (COVID-19), in Wuhan, Hubei province, China and rapidly outbroken into a major global pandemic [4,5,6]. It has been proved to have stronger infectivity but less virulence compared to SARS and MERS [7].

COVID-19 can manifest with a variety of symptoms from mild to severe (flu, fever, cough, fatigue, shortness of breath, infection of the lower respiratory tract, pneumonia, fibrosis with thrombosis in pulmonary small vessels, etc.) and even death (≈3.4%). It can also lead to complications associated with the immune response being out of control, such as disseminated intravascular coagulation (DIC) [8]. The severity of the disease depends on the efficiency of the affected individuals’ immune system and the presence of co-morbidities [9,10,11,12]. A common feature is the strong inflammatory response, which manifests through elevated C-reactive protein (CRP), pro-inflammatory cytokines production (Il-6, IL-10, IL-1), higher TNF-α, neutrophil count, D-dimer and blood urea [13]. SARS-CoV-2 spreads in the population at a rate of 0.8%–3% more than the normal flu and binds to angiotensin-converting enzyme 2 (ACE2) with high affinity to infect humans [14]. It mostly affects the elderly and people with chronic underlying diseases and it shows a preference for men [15], for reasons that we will discuss later.

At present, the only supporting treatments of CoV-19 flu are those aimed at the side effects caused by the virus—such as inflammation and pulmonary fibrosis, recognized as the first causes of death—symptomatic and respiratory support (oxygen therapy and extracorporeal membrane oxygenation) [9]. In some critical circumstances, convalescent plasma and immunoglobulin G have been administered to patients [16]. Several antiviral drugs and systemic corticosteroid treatment commonly used against influenza viruses are inefficient to treat COVID-19 [17]. Combinations of antiviral drugs, immunomodulatory, anti-parasite and common flu remedies have been tried with some results [18], but to date, scientists all over the world are working intensively on the therapies and vaccines against the virus.

As already mentioned, the spikes proteins of Coronavirus bind to ACE2 receptors, fusing to the cell membrane and releasing the viral RNA into the host cells. The viral RNAs are detected by Toll-like receptors (TLR) 3, TLR7, TLR8 and TLR9. Hence, virus–cell interactions produce a diverse set of immune mediators against the virus. Viral replication in host cells is always associated with inflammation and immune activation [19].

The immune system has complex mechanisms to fulfill its function and respond to a variety of signaling molecules including hormones, neurotransmitters, and specific lipids, such as endocannabinoids (eCBs) [20]. The biological effects of cannabinoids are mediated through the activation of G-protein-coupled cannabinoid (CB) receptors [21]. The endocannabinoid system (ECS) includes the cannabinoid receptor type 1 (CB1) and 2 (CB2), the endogenous cannabinoids, and the enzymes for their metabolism. CB1 is mostly expressed in the central nervous system and is strongly associated with the psychoactive effects of cannabinoids [22]. CB1 is also expressed at low levels in peripheral tissues [23]. Instead, CB2 is highly expressed by immune cells (B cells, natural killer cells, monocytes, neutrophils, CD8 lymphocytes, CD4 lymphocytes) [24,25] and in several organs and tissues such as liver, spleen, nasal epithelium, thymus, brain, lung and kidney [26,27,28]. Both CB1 and CB2 receptors have been widely demonstrated to be important modulators of the immune system, potentially inducing immunosuppression [29]. CB2 is widely known for its immunomodulatory role, which is related to four events: (i) induction of apoptosis, (ii) suppression of cell proliferation, (iii) inhibition of proinflammatory cytokines production and increase in anti-inflammatory cytokines and (iv) induction of regulatory T cells [30]. It is therefore conceivable that, also in COVID-19, the activation of the ECS plays a role in preventing and/or influencing the development and the severity of the disease.

D9-Tetrahydrocannabinol (D9-THC) and cannabidiol (CBD) are the phytocannabinoids that have been studied the most for their medicinal properties, due to their ability to suppress lymphocyte proliferation and inflammatory cytokine production [31,32,33]. However, they bind to both CB receptors; thus, considering that CB1 receptors are localized predominantly in the central nervous system, psychotropic effects have been often observed following their administration [23,24,25,26].

The specific activation of CB2 receptors induces apoptosis, inhibits the production of autoantibodies, pro-inflammatory cytokine expression, matrix metalloproteinases and bone erosion and induces a shift from a Th1 to Th2 immune response and induced myeloid-derived suppressor and T-regulatory cells [34]. In addition, CB2 receptor exerts an inhibitory effect on inflammatory processes [29], including macrophage migration [35], and provides an important therapeutic target for reducing some immune-pathological processes associated with viral infections [31,32,33].

Therefore, given the well-known involvement of CB2 receptors in immunomodulatory processes and the recent knowledge about the inflammatory, coagulative and cytokines misbalance that COVID-19 patients have to face, we describe the possible role of the CB2 receptor in modulating them, suggesting it as possible therapeutic target in COVID-19.

## 2. CB2 and Viral Infections

The immune system acts through complex mechanisms to accomplish its defensive function. Cells participating in the immune response bear cannabinoid receptors and in particular cannabinoid receptors type 2 [29]. Therefore, the activation of these receptors might have a decisive role in preventing and modulating the development of an infective disease. CB2 receptors are locally overexpressed in the presence of viral infection, and their activation through a selective agonist inhibits the leukocytes migration into the site of inflammation [36].

Many studies have examined the effect of cannabinoids on resistance to infections. Δ9-THC treatment seems to sensitize to several microbial infections [37], such as herpes simplex virus type 2, since Δ9-THC suppresses host defenses and, as well as the CB2 selective agonists, has suppressive effects on B-cells, monocyte/macrophages and dendritic cells. It is important to underline that compounds interfering with inflammatory processes could either compromise or improve the host response to viral infection because there are some viruses that benefit from host inflammation and other ones that are eradicated by host inflammation [38,39]. Agonists of CB2, but not CB1, have been shown to reduce infection in primary CD4+ T cells following cell-free and cell-to-cell transmission of CXCR4-tropic virus. CB2 agonist have been shown to decrease CXCR4-activation-mediated G-protein activity and MAPK phosphorylation, alter the cytoskeletal architecture of resting CD4+ T and impair productive infection following cell-free or cell-associated viral acquisition of CXCR4-tropic HIV-1 in resting cells. Thus, indicating that the clinical use of CB2 receptor agonists in the treatment of AIDS symptoms may also exert beneficial adjunctive antiviral effects against CXCR4-tropic viruses in late stages of HIV-1 infection [32]. CB2 stimulation also reduces some effects of inflammatory processes in HIV-infected patients [36]. HIV infection causes changes in CB2 receptor expression, as it has been observed during the process of in vitro monocyte differentiation into macrophages [40]. CB2 increases as HIV infection progresses, and on infected macrophages, the exposure to CB2 receptors selective agonist JWH133 resulted in a dose-dependent decrease of reverse transcriptase activity/viral replication activity [41].

Moreover, acute viral respiratory infections could be responsible for the onset of secondary bacterial super-infections, which cause a significant worsening of clinical course. The bacterial super-infection is caused by bacterial colonization of respiratory tracts damaged by the viral infection and, consequently, alteration of host immune responses [42]. SARS-CoV-2, in some cases, caused secondary bacterial infection, which worsened the prognosis [43]. Sepsis consists in both pro-inflammatory and immunosuppressive responses to an infection, which can induce multiple organ failure and death. The role of CB2 receptors in sepsis has been evaluated by Tschöp et al. who demonstrated that CB2 stimulation plays a key role in neutrophils. Its stimulation in fact reduced neutrophils number, decreasing mortality and tissue damage. A reduced neutrophil recruitment during sepsis is associated with increased survival. Moreover, neutrophils can kill bacteria and reduce tissue injury [44]. Therefore, the use of CB2 selective agonists could be suggested to regulate neutrophil recruitment and bacterial clearance. A variant of the CB2 receptor at codon 63 of the CB2 gene leads to the substitution of glutamine (Gln (Q)) with arginine (Arg (R)), with a consequent difference in protein polarization. These variants affect the response of CB2 receptor to cannabinoids. The receptor carrying R showed a reduced immune modulation function when activated by cannabinoids, therefore influencing the acquisition, the severity and the duration of the infection from other RNA viruses [45,46]. Cannabinoids may also induce less damage to endothelial barriers, thanks to their influence on several pro-inflammatory events [47]. We found that in HIV/HCV-coinfected patients, in T cells from QQ subjects, CB2 stimulation mediates the inhibition of their proliferation, while in subjects with the RR haplotype, T cells proliferation is reduced, indicating that the CB2-63RR variant is associated with weaker and transient inhibition of T cells compared to the CB2-63QQ. The CB2 RR variant has been also indicated as a prognosis worsener of liver necroinflammation in HIV/HCV-coinfected patients, while when it is caused by HCV monoinfection, the CB2 QQ variant is associated with more severe liver necroinflammation [48].

CB2 receptors activation has also been studied in children with viral respiratory infection using a selective agonist, JWH133. The CB2 Q63R variation was associated with a more severe clinical course of the acute viral infection and increased risk of hospitalization. Children infected with Respiratory Syncytial Virus carrying the QQ genotype showed the associated risk of developing severe respiratory complications increased more than two-fold. CB2 receptors activation by JWH133 reduced the cytokines production and limited lung pathology [41]. Collectively, CB2 receptor is associated with Respiratory Syncytial Virus infection severity during infancy, and it has been suggested as a therapeutic target to alleviate virus-associated immunopathology. Null mice for cannabinoid receptors show a greater inflammatory response to influenza infection, strongly suggesting that cannabinoid receptors have a role in immuneregulation [49,50].

Taken together, all those studies confirm that CB2 receptors have a central role in immune balance and negatively regulate the immune response magnitude. The immune system fights foreign agents, and the activation of CB2 receptors triggers anti-inflammatory action; therefore, targeting these receptors may be a novel and effective approach for the treatment of COVID-19.

## 3. SARS-CoV-2 and CB2 in Inflammation: Cytokines, Macrophages, Mesenchymal Stromal Cells

### 3.1. Inflammation and Cytokines Production

Among the clinical features of COVID-19 patients, there is a very high number of circulating inflammatory molecules, including C reactive protein (CRP) and pro-inflammatory cytokines [51]. In recent weeks, several authors observed and confirmed this alteration [52,53]; in particular, Huang et al. [54] measured cytokine levels in 41 patients reporting the increase of IL-1β, IL-7, IL-8, IL-9, IL-10, fibroblast growth factor (FGF), granulocyte-macrophage colony stimulating factor (GM-CSF), IFNγ, granulocyte-colony-stimulating factor (G-CSF), macrophage inflammatory protein 1 alpha (MIP1A), tumor necrosis factor (TNFα) and vascular endothelial growth factor (VEGF). The pro-inflammatory cytokine IL-6 seems to be critically high in severe COVID-19 patients. The altered cytokine profile observed in COVID-19 patients is very similar to the Cytokine Storm (CS) that characterizes SARS (severe acute respiratory syndrome) and MERS (Middle East respiratory syndrome), other two kinds of pneumonia caused by a coronavirus [55].

In the above-cited syndromes, CS and inflammatory cell infiltration in the lungs lead to severe injury, acute respiratory distress and death. Given the presence of CS also in COVID-19 patients, an anti-inflammatory therapy with non-steroidal anti-inflammatory drugs, glucocorticoids, cytokines antagonists, monoclonal antibodies (i.e., Tocilizumab, Anakinra, Idroxiclorochin, and others) or JAK inhibitors so far have proven to be helpful. On the other hand, the use of anti-inflammatory drugs could present some limits. First of all, cytokine inhibitors are available and specific only for a few of the cytokines actually involved in inflammatory cascade. About the use of corticosteroids, further investigations are needed; their capability to reduce both inflammation and immune response could be beneficial as well as could delay the elimination of the virus. But controversial hypotheses are present in literature regarding this issue. Chen Wang et al. [54] reported clinical evidence about SARS [56] and MERS [57], in which the administration of corticosteroids did not induce any difference in terms of mortality, but it was only associated with a worst clearance of viral RNA from the respiratory system. Moreover, therapies reducing immune response (such corticosteroids above mentioned) could increase the risk of new infection as well as fuel existing infections [58]. Indeed, patients treated with immunosuppressants are immunocompromised and therefore exposed to high hazard of mortality [59]. Puja Mehta et al. suggested, in fact, screening severe COVID-19 patients for hyperinflammation (considering ferritin levels, platelet count, erythrocyte sedimentation rate, etc.) to identify individuals in which immunosuppression could be fatal [60].

Therefore, there are yet discordant opinions about the most suitable treatment. In a multicenter study on 150 COVID-19 patients in Wuhan, Ruan Q. et al., showed that in addition to the overproduction of inflammatory cytokines, especially of IL-6, there is also an increase in ferritin levels [61]. These data suggest a virus-dependent hyperinflammation in which the immunosuppressive effect of anti-inflammatory drugs could be beneficial instead. In 2016, Shakoory B. et al. highlighted this effect of the IL-1R antagonist (Anakinra) in reducing mortality in patients with macrophage activation syndrome [62]. These authors then suggest screening the severe COVID-19 patients for hyperinflammation and identify those who could benefit from the immunosuppressive effect of anti-inflammatory therapy.

The role of the endocannabinoid system in modulating inflammation is well known, and in particular, in cytokine release. In the literature, it is reported that AEA, an endogenous agonist with high affinity to CB1, reduces the production of pro-inflammatory IL-6 [63], and it is known that THC, a CB1 and CB2 receptor partial agonist inhibits the release of IL-12 and IFN-γ [64]. Moreover, in 2014, Sardinha et al. demonstrated in vivo that the inhibition of MAGL and FAAH, the enzymes that respectively degrade 2-AG and AEA, has CB2-mediated anti-inflammatory effects [65]. Also (E)-β-caryophyllene ((E)-BCP) is a phytocannabinoid that selectively binds to the CB2 receptor, and it is a functional CB2 agonist. (E)-BCP inhibits lipopolysaccharide (LPS)-induced proinflammatory cytokine expression in peripheral blood and attenuates LPS-stimulated Erk1/2 and JNK1/2 phosphorylation in monocytes. Furthermore, (E)-BCP administration strongly reduces the inflammatory response in wild-type mice but not in mice lacking CB2 receptors, providing evidence that this natural product exerts cannabimimetic effects in vivo. These results identify (E)-BCP as a functional non-psychoactive CB2 receptor ligand and as an anti-inflammatory cannabinoid. (E)-BCP has effects also on vascular inflammation and significantly ameliorated vascular oxidative stress [66,67].

Furthermore, there are several pieces of evidence about the specific involvement of CB2 receptor in modulating inflammation in different pathologies. To begin, in 2015, Verty AN et al. [68] observed that JWH-015, a CB2 receptor agonist, reduced obesity-associated inflammation in mice. The next year, 501 Italian obese children were genotyped for the CB2 Q63R variant, a less functional variant of CB2, highlighting that this variant was associated with high levels of pro-inflammatory IL-6 similar to the levels observed after blocking CB2 receptor in lean-derived adipocytes in vitro [69]. This and many other alterations seem to contribute to the low-grade inflammation of white adipose tissue in obese people [70]. The same CB2 Q63R variant was associated also with liver necroinflammation in chronic hepatitis patients with HIV/HCV coinfection [48], synovium inflammation in juvenile idiopathic arthritis [71], liver damage in children with non-alcoholic fatty liver disease [72] and inflammation of gastro-intestinal tract in inflammatory bowel disease (Crohn’s disease and ulcerative colitis) [73] and in celiac disease [74]. Moreover, it has been demonstrated that the cannabinoid CBD inhibits the production of the pro-inflammatory cytokines IL-6, IL-8 and TNF-α in in vitro models of allergic contact dermatitis [75], and in osteoarthritis, THC reduced TNF-α, IL-1β, IL-6 and IL-8 release in LPS-stimulated MG63 cells, demonstrating the anti-inflammatory CB2-mediated role [76].

Immune thrombocytopenia (ITP) is another disease characterized by abnormal cytokine secretion and influenced by the presence of the CB2 Q63R variant. In particular, mesenchymal stem cells from ITP patients overproduce the pro-inflammatory cytokine IL-6. Regular levels are restored using JWH-133, a selective agonist at CB2 receptors [77]. A proper activation of CB2 receptor reduces the levels of several inflammatory mediators (IL-6, IL-1β and TNF-α) also in animal model of multiple sclerosis [78].

### 3.2. Inflammation and Macrophages

The importance of macrophages’ role in SARS-CoV-2 infection has been assessed by demonstrating a crosstalk between macrophages and the ACE2-expressing cells in lung, liver and stomach. Macrophages are recruited by CoV-targeted cells during inflammation, and they play a defensive or destructive role in infection [79]. In particular, it has been demonstrated that in lungs of COVID-19 patients with diffused alveolar damages, the cell infiltration consists mainly of macrophages and monocytes, moderate mononuclear giant cells and very few lymphocytes. After virus infection, those cells are responsible for the “primary cytokine” storm mentioned above [55]. The presence of inflammatory cells infiltration is responsible for acute lung injury, causing acute respiratory distress syndrome and death [80].

Macrophages are mononuclear phagocytes with a key role in inflammatory response, cytokines production, phagocytosis, cellular proliferation and tissue restoration in wounds. They are characterized by a remarkable plasticity, showing two different activation phenotypes based on the microenvironment in which they lay [81]: classically activated macrophages (M1) and alternative activated macrophages (M2). M1 macrophages are activated after interferon-gamma (INF-γ) and lipopolysaccharide (LPS) stimulation. They exhibit pro-inflammatory and anti-tumor properties by releasing various types of pro-inflammatory cytokines and chemochines, such as Tumor Necrosis Factor (TNFα), Interleukin-6 (IL-6), Interleukin-1 Beta (IL-1β) and Nitric Oxide Synthase (INOs). On the other hand, M2 polarization is promoted both by Phosphatidylinositol 3-kinase-AKT-mammalian target of rapamycin (PI3K-Akt-mTOR) signaling pathway and by the anti-inflammatory cytokines Interleukin-4 (IL-4) and Interleukin-10 (IL-10); they perform anti-inflammatory and immunosuppressive effects by releasing anti-inflammatory cytokines (IL-10) and promote tumor progression. An imbalance of M1/M2 is responsible of inflammation [81,82,83].

It is known that CB2 receptors are mainly expressed in peripheral immune cells, including macrophages [20]. Several studies have demonstrated a role for this receptor as a mediator of anti-inflammatory and immunosuppressive properties. It inhibits immune cell activation and pro-inflammatory mediator release (cytokines, reactive oxygen species (ROS), nitric oxide, etc.). Thus, it has been suggested as a possible target for treatment of inflammatory and autoimmune diseases, such as inflammatory bowel disease, juvenile idiopathic arthritis, inflammatory bowel disease, celiac disease, obesity and neuroinflammatory diseases [84,85]. All these pathologies are characterized by an alteration of immune cell activation and an increase of pro-inflammatory cytokines release.

Moreover, it has been shown that CB2 receptor stimulation with its selective agonists reversed these pathological conditions by reducing both B and T lymphocyte [86], by promoting mesenchymal stromal cells’ (MSCs) homing and immunosuppressive and anti-inflammatory activities [77,87] and by limiting pro-inflammatory cytokine release in macrophages, inhibiting M1 polarization [83].

Several studies have highlighted the importance of the role of CB2 receptors as regulators of macrophage polarization in inflammatory processes. In particular, it has been shown that its stimulation with selective agonists induced a reduction of the pro-inflammatory macrophage population (M1) and an increase of the anti-inflammatory phenotype (M2) [88]. Du et al. have demonstrated that stimulation of CB2 receptor with its selective agonist JWH-133 attenuated inflammation during skin wound healing by inhibiting M1 macrophages rather than by activating M2 macrophages in skin lesion. They showed a significant reduction of M1 markers and pro-inflammatory cytokines, CD86, iNOS, IL-6 and IL-12, after treatment with JWH133 or GP1a. These results indicated that CB2 inhibited the release of pro-inflammatory cytokines, preventing the macrophages polarization to the M1 phenotype [83].

Also in neuroinflammation, CB2 receptor stimulation exerts its anti-inflammatory effects, modulating macrophage polarization. Braun et al. demonstrated that, in patients with neuroinflammation induced by traumatic brain injury, stimulation of CB2 receptor with its selective agonist, GP1a, induced M2 anti-inflammatory macrophage polarization and inhibited M1 pro-inflammatory polarization, determining a reduction of pro-inflammatory mediator expression (TNFα, IL1β, IL6, CCl2, CXCL10 and iNOS) and an increase of anti-inflammatory mediator expression (IL10, ArgI) [88].

CB2 receptor displays its anti-inflammatory properties also in alcoholic liver disease, by acting on Kupffer cells polarization. Louvet et al. proposed CB2 receptors as a novel regulator of Kupffer cell polarization. Their in vivo and in vitro experiments showed an increase of M1 phenotype markers and a reduction of the M2 phenotype markers in response to chronic alcohol feeding after genetic deletion of CB2. Instead, after JWH-133 treatment, they observed an inhibition of pro-inflammatory M1 profile by shifting the M1/M2 balance toward a predominant alternative M2 response, and a reduction of inflammation [26]. Moreover, human lung-resident macrophages express CB2 receptor, and its stimulation induces a reduction in the release of some pro-inflammatory cytokines (such as IL-6) and angiogenic factors [89].

### 3.3. Mesenchymal Stromal Cells (MSCs) in Inflammation

In COVID-19 patients, an alteration in cytokine production is present that is very similar to the process called cytokine storm, characterized also by an overproduction of immune cells [59]. Considering the well-known anti-inflammatory function of mesenchymal stromal cells (MSCs) [90,91], in the last few months, several authors investigated the possibility to use MSCs to treat COVID-19 patients. In particular, these cells seem to reduce the secretion of inflammatory factors, thus improving lung function after acute injury caused, for example, by influenza virus. Jiajia Chen et al. [92] performed a clinical study in which they tested menstrual-blood-derived MSCs in patients with acute respiratory distress syndrome (ARDS) caused by H7N9 infection and observed benefits in the most severe cases.

H7N9 is a subtype of influenza A viruses with symptoms very similar to COVID-19 (cough, fever, shortness of breath, etc.) and with similar complications (ARDS and lung failure) [93,94]. Hence, the authors suggested that a therapeutic strategy used to manage H7N9 inflammatory damages could be used also in ARDS-induced severe pneumonia of COVID-19 patients. In detail, MSCs have the capability to increase the number of peripheral lymphocytes and at the same time to reduce the cytokine-secreting immune cells (CD4+ T cells, CD8+ T cells and NK cells) in the circulating blood [95,96] without any adverse reaction [97]. This immunomodulatory effect is due to their interaction with immune cells, directly or mediated by paracrine cytokines [98,99]. Beyond the great influence that MSCs exert on immune response, it has also been observed that they produce a specific molecule, the leukemia inhibitor factor (LIF), useful in counteracting the cytokine storm in viral pneumonia [100]. The LIF amount produced by MSCs is not enough, but in literature is reported the use of synthetic stem cells (LIF-Nano) with a 1000-fold greater potency in producing LIF and able to reverse paralysis in preclinical model of multiple sclerosis within 4 days [101].

**Figure 1:**
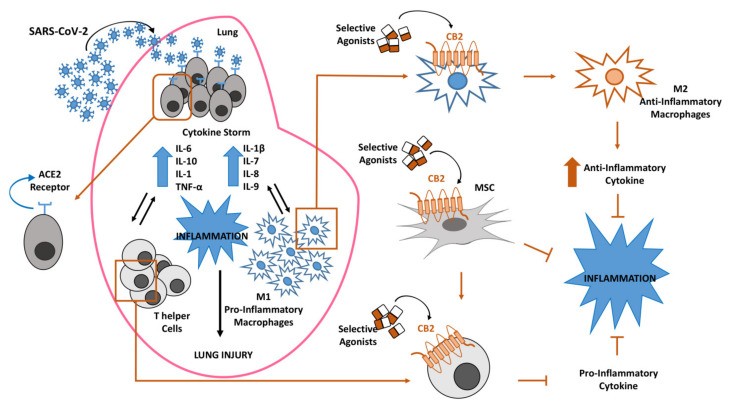
Inflammatory response in lung after coronavirus (SARS-CoV-2) infection. Lung susceptibility to SARS-CoV-2 infection depends on viral spike proteins specificity for angiotensin-converting enzyme 2 (ACE2) receptors on alveolar epithelial cells. This interaction leads to hyperinflammation sustained by cytokine storm, increase of pro-inflammatory M1 macrophages and T-helper cells, all associated in a vicious circle in which each event enhances the alteration of the other ones. The selective stimulation of Cannabinoid Receptor type 2 (CB2) receptors on macrophages, T-helper cells and mesenchymal stromal cells (MSCs) could be proposed to contain the inflammatory state in COVID-19 patients.

From our previous studies, we know that MSCs abundantly express CB2 receptors and that this feature, together with the above-described characteristics, makes them suitable in managing CoV-19 infection. It has been observed that the selective stimulation of MSCs with agonists at CB2 receptor, JWH-133, improved their survival and their immunomodulating properties with important impact in regulating lymphocytes activity and cytokine secretion [77]. On these bases, MSCs therapy, together with a proper stimulation of their CB2 receptor, could be proposed to improve COVID-19 patients’ conditions with a double function: to repair tissue damages on stem cells and to drive immune response in a protective direction immunomodulating cells. MSCs are easy to access and isolate from different sources (umbilical cord, dental pulp, menstrual blood, etc.), and they can be stored for repetitive therapeutic usage with absolute effectiveness [102,103,104]. Moreover, with RNA-sequencing, it has been observed that MSCs are negative for ACE2 and TMPRSS2 [105], the main proteins involved in COVID-19 pathogenesis; therefore, these cells can be safely infused in affected patients without being infected by CoV-19 rather bringing all the above-mentioned beneficial effects to the host [106] (Figure 1).

## 4. CB2 and Estrogens

Several epidemiological studies suggest sex-specific differences in the incidence of CoV-19/SARS-CoV-2, with men more susceptible to infection (about 70% of infected patients) than women [15,52,54]. Interestingly, this difference has already been observed in the past for other viral infections such as severe acute respiratory syndrome (SARS)-CoV and Middle East respiratory syndrome (MERS)-CoV [107,108]. In effect, it is already widely known that males and females react differently to RNA virus infections [64]. In general, males respond with a less strong immune response [109]. Women are less susceptible to viral infections for various reasons related to a different innate immunity, sex chromosomes [110] and especially steroid hormones [111]. Female hormones seem to confer a natural resistance against many diseases. At high concentrations, estrogens have an immune-suppressive effect and at low concentrations exhibit an important immune-stimulatory activity [112]. Steroid hormones exert their effects through intracellular receptors that can regulate the expression of target genes by binding to specific enhancer elements [113]. The role of estrogens in modulating cannabinoid receptor expression and endocannabinoids levels is widely known, both in physiological and in pathological conditions [114,115,116]. Studies have demonstrated that 17β-estradiol increases the expression of CB2 receptors in osteoclasts in vitro through the recruitment of an estrogen-responsive element in the CB2 gene [117]. In addition, selective estrogens receptor modulators (raloxifene, bazedoxifene and lasofoxifene) act as CB2 receptors agonists [118,119]. Estrogens and cannabinoids share several molecular pathways and involvement in several inflammatory processes [120]. Peretz et al. demonstrated a role of estrogens in inhibiting influenza A virus replication in nasal epithelial cells derived from humans [121]. Accordingly, Channappanavar et al. showed a protective effect of estrogen signaling in mice infected with SARS-CoV-1, demonstrating that the ovariectomy or pharmacological antagonism of estrogen receptor in female mice increases mortality [122]. Moreover, they observed a large number of macrophages and an increased level of pro-inflammatory cytokines in the lungs of SARS-CoV1–infected ovariectomized mice compared with control female mice, suggesting that estrogen signaling is able to suppress macrophage activity in the lungs, probably through the NF-κB inhibition and the subsequent pro-inflammatory cytokine production [122]. Considering that the CB2 receptor regulates the immune system and inhibits inflammation in many inflammatory disease [85], it is conceivable that the protective effects of estrogens could strongly be related to a CB2 receptor activation. In a model of lung injury, CB2 receptor up-regulation inhibits NF-κβ activity, reducing pro-inflammatory factors release (TNF-α, IL-12 and IL-6) and increasing anti-inflammatory factors (IL-10 and IL-4) production [123], confirming that CB2 receptors activation may act as a novel immunomodulatory strategy to alleviate lung diseases through the inhibition of immune cells.

## 5. Conclusions

We have discussed the clinical features of SARS-CoV-2 infection, including the severe acute inflammation that causes cytokine storm in COVID-19 patients.CB2 receptors stimulation is known to exert anti-inflammatory and immunomodulating effects by reducing the release of pro-inflammatory cytokines, by shifting the M1/M2 ratio towards the anti-inflammatory M2 macrophage phenotype and by improving the MSCs-repairing properties. It is also well documented that human lungs, macrophages and MSCs, express CB2 receptors. Estrogens exert a protective effect in COVID-19, which explains sex-specific differences observed in SARS-CoV-2 infection. This could also be related to a CB2 activation. We suggest therefore, the possibility of using CB2 as a pharmacological target for the treatment of SARS-CoV-2 infection.

We hypothesize that the selective stimulation of CB2 could reduce the inflammatory response in SARS-CoV-2 patients and could improve the outcome. The stimulation of CB2 could control the inflammatory cascade in several checkpoints, considering its capability to reduce the production of a large number of cytokines, contrarily to the extremely selective action of monoclonal antibodies directed against a specific interleukin. On the other hand, CB2 receptor stimulation has a well-documented immunosuppressive effect by reducing immune cells proliferation [124] and production of antibodies [125]; thus, it could be greatly beneficial in containing the exacerbated inflammatory response in COVID-19 patients.

To date, there are no commercially available agonists, approved for the use in human subjects, that specifically bind to CB2 receptors. HU910, HU308 and JWH133 have high specificity to CB2 receptors and are recommended to study the role of this receptor in biological processes and diseases [126]. Cannabidiol (CBD) is also involved in modulation of inflammatory processes through a CB2-dependent mechanism. It induces CB2 activation indirectly, by increasing AEA levels, and exerts its anti-inflammatory properties by reducing pro-inflammatory cytokines release in experimental model of allergic contact dermatitis [127]. A novel ∆9-tetrahydrocannabinol (∆9-THCP) binds with high affinity to both human CB1 and CB2 receptors. In particular, the affinity shown for CB1 is thirty-fold higher compared to the one reported for Δ9-THC in the literature, and it was 5 to 10 times more active on the CB2 receptor. It has also been demonstrated that Δ9-THCP showed a cannabimimetic activity several times higher than its pentyl homolog Δ9-THC, also at lower doses [127]. Nevertheless, more studies are necessary to develop a commercially available CB2 selective agonist, and clinical studies with the available phytocannabinoids should be encouraged.

Another interesting field of investigation could be the screening of COVID-19 patients for CB2 Q63R. In this way, it would be possible to clarify if, also in this case, the variant is a predisposing factor to the infection and also if it is associated with the appearance of the most severe side effects (respiratory distress, pulmonary fibrosis and death). All these actions could produce better knowledge on SARS-CoV-2 pathogenesis and significantly improve the management of COVID-19 patients.
